# Applying Theory of Planned Behavior to Understand Physicians’ Shared Decision-Making With Patients With Acute Respiratory Infections in Primary Care: A Cross-Sectional Study

**DOI:** 10.3389/fphar.2021.785419

**Published:** 2022-01-27

**Authors:** Dan Wang, Xinping Zhang, Haihong Chen, Chenxi Liu

**Affiliations:** ^1^ School of Management, Hubei University of Chinese Medicine, Wuhan, China; ^2^ School of Medical Management and Health Management, Tongji Medical College of Huazhong University of Science and Technology, Wuhan, China; ^3^ School of Health Policy and Management, Nanjing Medical University, Nanjing, China

**Keywords:** shared decision-making, theory of planned behavior, physicians, patient with acute respiratory infections, primary care

## Abstract

**Background:** To understand the physicians’ shared decision-making behavior (SDM) with patients with acute respiratory infections (ARIs) based on the theory of planned behavior (TPB) and identify barriers to the implementation of SDM in primary care.

**Methods:** A cross-sectional study of 617 primary care physicians was conducted in primary facilities in Hubei province, China from December 2019 to January 2020. A self-administered questionnaire based on TPB theory was applied for measuring the physicians’ SDM behavior with patients presenting with ARIs.

**Results:** The proposed TPB model revealed that attitude and subjective norms predicted behavior intention, and behavior intention was one significant predictor of SDM behavior (*p* < 0.001). After controlling for physicians’ demographic characteristics, receiving training regarding antibiotics was significantly associated with physicians’ attitudes toward SDM, while educational level and gender were significantly associated with physicians’ intention of engaging in SDM (*p* < 0.05). Physicians’ perceptions of patients’ expectations and incapability of making decisions were the most frequently reported barriers to the implementation of SDM.

**Conclusion:** The TPB theory provides insights for understanding physicians’ SDM behavior with patients with ARIs in primary care. Since attitudes, subjective norms, and behavior intention were demonstrated as significant predictors of SDM behavior, these may be a promising focus of SDM interventions based on TPB theory. The results of the TPB model and potential barriers of SDM behavior would help determine future directions for SDM training and educating the public.

## Introduction

In recent years, with the rapid development of patient-centered care and the increasing demand of patient participation in medical care, shared decision-making (SDM) is recommended as a patient-centered approach in which the health-related decision-making process is made jointly by the patient and healthcare providers (Charles et al., 1997; Epstein et al., 2005; Elwyn et al., 2012). Health organizations, health scholars, and healthcare providers advocated SDM as a strategy for improving the physician–patient relationship and optimizing clinical outcomes (Barry and Edgman-Levitan, 2012; Härter et al., 2017).

In primary care facilities of China, the setting of this study, there is around 50% of outpatient consultation in primary care facilities involved an antibiotic prescription (Liu et al., 2019), but less than 40% were appropriate prescriptions (Wang et al., 2014). Acute respiratory infections (ARIs) are one of the most common reasons for visits to primary care physicians and antibiotics prescription (Costelloe et al., 2010; WHO, 2014). For most ARI cases, the decision about whether to treat with antibiotics or not is nearly at equipoise (Bakhit et al., 2018). The shared decision process is especially advocated for such preference-sensitive decisions, in which the SDM process promotes shared medical decisions and more appropriate antibiotic use in primary care (Coxeter et al., 2015; Trivedi, 2016; Barreto and Lin, 2017; van Esch et al., 2018).

However, how to integrate SDM into daily practice remains a major challenge, such as challenges of operationalizing and measuring SDM, and identification of effective tools facilitating SDM (Joseph-Williams et al., 2014; Joseph-Williams et al., 2017; Hawley, 2018). For example, physicians feel they already involved the patient in treatment decisions, or they can serve as delegates for patients in decisional process (Joseph-Williams et al., 2017). To help understand SDM and integrate SDM in clinical practice, a thorough understanding of physicians’ SDM behavior and the determinants that underlie the behavior is of great significance and would help determine future directions for SDM training and educating the public.

To enable the understanding of physicians’ SDM behavior, the theory of planned behavior (TPB) is considered appropriate with a great predictive performance of behavioral intention and performance (Ajen, 1988; Armitage and Conner, 2001; Godin et al., 2008). Philippe et al. reviewed physicians’ SDM behavior studies based on the TPB framework; however, the majority of studies were conducted in developed countries with a focus on the general judgment of SDM. Little was known regarding the physicians’ SDM behavior and its influencing factors under the specific context with patients presenting with ARIs (Thompson-Leduc et al., 2015).

Several studies have observed differences in SDM behavior between different groups of physicians (Brinkman et al., 2011; Sonntag et al., 2012). Based on previous researches, physicians with different ages, gender, professional titles, and duration of working years may have different attitudes or SDM behavior. However, the results were mixed; for example, physicians with different age groups and gender showed inconsistent results toward the perception of SDM (Brinkman et al., 2011; Sonntag et al., 2012; Alameddine et al., 2020). The mixed results deterred identification of the effects of different physician groups on SDM for future interventions, and thus further subgroup analysis is needed.

To support the future implementation of SDM and promotion of the rational use of antibiotics in primary care in China, our study aims to understand the physicians’ SDM behavior based on TPB in patients presenting with ARIs in primary care and whether the relationship of SDM behavior differs between subgroups of physicians. On the other hand, to supplement the TPB results, potential barriers to the implementation of SDM will also be explored in primary care.

### Theoretical Framework

The theory of planned behavior (TBP) framework was adopted in this study. Attitudes (favorability to perform a behavior), subjective norms (perceived social pressure to perform a behavior), and perceived behavioral control (perceived ease or difficulty to perform a behavior) in relation to SDM are linked with physicians’ SDM behavior.

The TPB model assumed that attitudes, subjective norms, and perceived behavioral control can influence behavioral intention, and the SDM behavior is influenced by behavior intention and perceived behavioral control (Armitage and Conner, 2001).

### Hypotheses

According to the TPB, the following hypotheses were examined in this study with patients presenting with ARIs.

H1: Physicians’ attitudes toward SDM positively impacted physicians’ intention to engage in SDM;

H2: Physicians’ subjective norms positively impacted physicians’ intention to engage in SDM;

H3: Physicians’ perceived behavioral control positively impacted physicians’ intention to engage in SDM;

H4: Physicians’ intention to engage in SDM positively impacted physicians’ SDM behavior;

H5: Physicians’ perceived behavior control positively impacted physicians’ SDM behavior.

## Materials and Methods

### Study Setting and Sampling

A cross-sectional study was conducted in Hubei, a province with a middle level of social and economic development in central China (National data National Bureau of Statistics of China). The study was conducted in primary care sectors covering urban community health centers (CHCs) and rural township health centers (THCs). There were 1,161 CHCs and 1,139 THCs, serving 43.53 million outpatients in Hubei province in 2018 (National data National Bureau of Statistics of China).

A two-stage cluster sampling was applied in this study, which was described in detail and published in one recent research conducted by the research team (Wang et al., 2020). The first stage involved a random selection of five cities (the provincial capital Wuhan and four prefecture-level cities in Hubei province). The second stage involved one urban district and one rural county selection in the selected five cities. All the primary care facilities in each selected city/county were investigated from December 16, 2019 to January 17, 2020. The physician who were licensed to prescribe antibiotics and who had authorized ≥100 prescriptions over the past 3 months prior to the survey were eligible for this study. A total of 779 physicians were eligible for this study. Finally, 617 eligible questionnaires were collected, giving a response rate of 79.2%.

### Measurements

A self-administered questionnaire was applied in this study. The TPB model assessed attitudes, subjective norms, perceived behavior control, behavior intention of SDM, and physicians’ SDM behavior. The specific measurements were presented as follows. In addition, the barriers to the implementation of SDM were measured to supplement the results of TPB model (Section 2.5).

#### The TPB Model

Based on the TPB model, our self-administered questionnaire assessed physicians’ attitudes, subjective norms, perceived behavior control, and behavior intention of SDM when a treatment decision needs to be made during a consultation with a patient with ARIs. All the items were responded with a Likert-5 scale. A higher score represented a more favorable attitude, higher pressure, higher perceived behavior control, and higher behavior intention.

Considering that busy physicians are reluctant to complete long questionnaires, our research team designed the questionnaire with limited length by validated items based on previous researches and guidelines for the TPB survey (Francis et al., 2004; Guerrier et al., 2013). The list of items in this study is presented in Supplementary Material S1.

#### Attitudes (3 Items)

Attitude refers to the degree to which a physician being in favor toward the SDM based on its potential outcomes. Physicians were asked to rate the degree of value, appropriateness, and the responsiveness of SDM.

#### Subjective Norms (4 Items)

Subjective norm refers to a physician’s perceived social pressure to engage in the SDM or not. Physicians were asked to rate the degree of pressure from the person who is important to the physicians (e.g., peers, patients, or administrators).

#### Perceived Behavioral Control (3 Items)

Perceived behavioral control refers to the perception of hindering or facilitating factors, and reflects personal resources to be able to engage in SDM. Physicians were asked to rate the degree of capability, ease, or difficulty for physicians to engage in SDM.

#### Behavior Intention (2 Items)

Behavior intention refers to the willingness of physicians to engage in SDM. Physicians were asked to rate the degree of physicians’ wants and expectations to engage in SDM.

#### Physicians’ SDM Behavior (2 Items)

The first item measured physicians’ decision-making role based on the Control Preference Scale (Degner et al., 1997). Five scenarios concerning the decisional approach with patients with ARIs were designed for each physician. Physicians were asked which of the five scenarios best exemplifies their approach in clinical practice with patients presenting with ARIs (Table 1).

**TABLE 1:** Physicians’ actual decision-making role based on the Control Preference Scale

*Paternalistic way*	a. I make the final decision about the treatment or further investigations
	b. I make the final decision about the treatment or further investigations, after seriously considering patient’s opinion
*Informative way*	c. The patient makes the final decision about the treatment or further investigations
	d. The patient makes the final decision about the treatment or further investigations, after seriously considering my opinion
*Shared decision-making*	e. Together, I and the patient share the responsibility for deciding the final treatment decision or further investigation

The second item aimed to quantify the proportion of SDM in actual medical encounters: “What is the percentage of your engagement in shared decision-making in actual medical encounters with patients presenting with ARIs?” The scores ranged from 0 to 100% and were classified into five categories of 0–20%, 20–40%, 40–60%, 60–80%, and 80–100%.

### Pilot Study

The questionnaire was piloted to evaluate the readability and reliability of the self-administered questionnaire. The questionnaire was tested using a sample of 30 primary care physicians in urban community health centers in Wuhan. Physicians were asked to complete the questionnaire and provide verbal feedback regarding the item’s readability. Some phrases were reworded according to the verbal feedback.

### Data Collection Procedure

The trained research teams were paired and dispatched to the selected CHCs and THCs. The physicians who were licensed to prescribe antibiotics and the researchers administered the TPB survey to the physicians. Physicians’ demographic and work characteristics, such as professional titles and working years, were also collected.

### The Barriers to the Implementation of SDM

The barriers to the implementation of SDM were designed to supplement for the results of the TPB with physicians’ perceived specific barriers to the implementation of SDM.

At the end of the TPB survey, physicians were asked to rate one or more barriers to the implementation of SDM in primary care. Several options were provided based on previous researches, such as lack of time or physicians’ perceived responsibility for making decisions for patients (Driever et al., 2020; Mathijssen et al., 2020). In addition, physicians are allowed to give answers outside the given options.

The TPB survey was administered in Chinese to the participants. As the original items from previous TPB studies and the Control Preference Scale were English written, a double translation was applied to ensure the consistency of translation, namely, the questionnaire was translated into Chinese and back-translated into English.

### Covariates

Some studies have reported associations between primary care physicians’ age or gender and their SDM behaviors (Brinkman et al., 2011; Sonntag et al., 2012). Also, clinical knowledge helps physicians weigh the treatment options and increase accuracy of risk perception, and physicians with different professional titles (such as resident physicians and senior physicians) and duration of working years may have different attitudes toward SDM based on previous studies (Alameddine et al., 2020; Oerlemans et al., 2021). These factors were considered to affect the physicians’ SDM behavior in this study.

Physicians’ age, gender, educational level, professional titles, working years, and receiving training in the past year were collected. The physicians were divided into subgroups based on the key characteristics in the TPB model. The professional titles were classified into three levels: resident physicians, attending physicians, and associate or chief physicians. Educational level was classified into senior high school and below, university degree, and graduate degree. The specific situation of medical education in China needs attention. Medical graduates with a non-bachelor degree can become assistant doctors. After accumulation of working experience, and if they pass an examination, they have the chance to get the full doctor license (Degner et al., 1997).

In addition, the key demographic and work characteristics were adjusted in the TPB model as covariates. The hypotheses of the effects of covariates on the TPB model are presented in Supplementary Material S2.

#### Statistical Analysis

The Statistical Package for Social Sciences for Windows (SPSS, version 22.0; Armonk, NY) and MPLUS (version 8.0; Los Angeles, CA) were jointly used to conduct the statistical analyses. Descriptive data were statistically analyzed using χ^2^ and Mann–Whitney U tests.

Prior to establishing the physician’s behavior model, the reliability and validity of the TPB survey were determined in the psychometric testing phase by calculating Cronbach’s alpha and factor analysis. The reliability with Cronbach coefficient alpha values for each construct of SDM behavior was 0.89 (attitudes), 0.78 (subjective norms), 0.65 (perceived behavior control), and 0.83 (behavior intention) in the main study. The confirmatory factor analysis test results showed good model fit indices of the expected four-factor model based on TPB theory with TLI = 0.98, CFI = 0.99, and RMSEA = 0.078.

The structural equation model was applied to understand the associations of SDM behavior and its determinants based on the TPB theoretical framework.

Since the responses were ordinal variables, means and variance adjusted weighted least squares extraction estimation was applied to examine the direct and indirect associations among the study variables.

### Evaluation of Model Fit

Goodness-of-fit indices were applied to evaluate the fit of the structural equation model: Tucker–Lewis index (TLI; >0.90 acceptable, >0.95 excellent), the comparative fit index (CFI; >0.90 acceptable, >0.95 excellent), root mean square error of approximation (RMSEA; <0.08 acceptable, <0.05 excellent), and weighted root mean residual (WRMR; <1.0 acceptable, <0.9 excellent) (Bentler, 1990; Hu and Bentler, 1999).

## Results

### Demographic Results

A total of 617 primary care physicians were investigated in the main study. The majority of physicians have a university or graduate degree (83.1%), while only a small proportion of physicians held senior job titles (13.0%). The median working years of the physicians was 16 years, and over 80% of physicians received training regarding antibiotics last year. The demographic and work characteristics of the physicians are presented in Table 2.

**TABLE 2:** Demographic and work characteristics of the physicians (*n* = 617)

Characteristics	N (%)/median (IQR)
Gender	
Male	389 (63.00)
Female	228 (37.00)
Age	44.0 (38.0–49.0)
Facility setting	
Community health center	220 (35.70)
Township health center	397 (64.30)
Educational level	
Senior high school and below	104 (16.90)
University degree	499 (80.80)
Graduate	14 (2.30)
Professional title*	
Resident physicians	302 (49.00)
Attending physicians	234 (38.00)
Associate or chief physicians	80 (13.00)
Annual household income (yuan)*	
<40,000	122 (20.00)
40,000**–**79,999	277 (45.30)
80,000**–**119,999	138 (22.60)
≥120,000	75 (12.10)
Years of clinical practice (years)	16.0 (9.0–24.0)
Training regarding antibiotics last year	
Yes	502 (81.40)
No/do not know	115 (18.60)
The actual role of decision-making	
Paternalistic role	241 (39.06)
Informative role	90 (14.59)
Shared decision-making role	286 (46.40)
The proportion of physicians engaging in SDM*	
0–20%	117 (19.8)
20–40%	96 (16.2)
40–60%	138 (23.4)
60–80%	171 (28.9)
80–100%	69 (11.7)

IQR, interquartile ranges (25th to 75th percentile). *There were missing cases.

### Measurement Score of Physicians’ Perceptions and SDM Behavior

The physicians’ attitudes showed a strong inclination in favor of the SDM (mean = 3.92, SD = 0.70). They felt relatively high social pressure (subjective norms) of engaging in SDM (mean = 3.34, SD = 0.73) and their perception of behavioral control of engaging in SDM was positive (mean = 3.26, SD = 0.55). In addition, the physicians reported a relatively high intention to engaging in SDM (mean = 3.62, SD = 0.85) in primary care.

Most primary physicians (46.4%) reported SDM as their actual decision-making role with patients with ARIs, while 39.06% of physicians preferred the paternalistic approach when dealing with the decision-making with patients presenting with ARIs. As for the actual proportion of participation in SDM, only 19.8% of physicians reported they never or seldom engaged in SDM, while 11.7% of physicians reported that they engaged in SDM in more than 80% of medical encounters with patients presenting with ARIs (Table 2).

### Structural Equation Model for SDM Behavior

**FIGURE 1:**
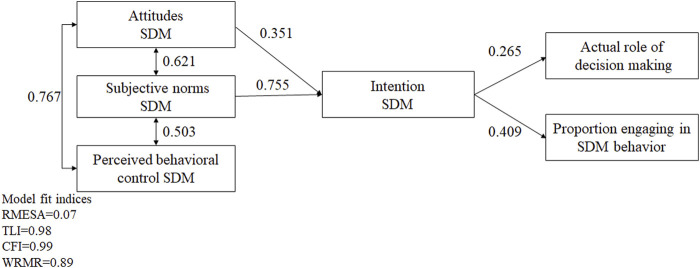
The TPB theoretical framework for shared decision-making with patients with ARIs. Figure 1 describes the results of five hypotheses of shared decision-making based on the theory of planned behavior.

The final structural equation model had an excellent fit to these data (TLI = 0.98, CFI = 0.99, RMSEA = 0.07, WRMR = 0.89) and the detailed information is presented in Figure 1. Attitudes and subjective norms of SDM were identified as significant predictors of behavior intention, and behavior intention was demonstrated as one significant predictor of physicians’ SDM behavior (*p* < 0.001).

As initially hypothesized, attitudes toward SDM (*β* = 0.351, *p* < 0.001) and subjective norms (*β* = 0.755, *p* < 0.001) had direct effects on SDM behavior intention. As for the linkage between SDM behavior intention and SDM behavior, the higher intention of SDM will contribute to a more collaborative or active role with medical decisions (*β* = 0.265, *p* < 0.001) and a higher proportion of physicians engaging in SDM (*β* = 0.409, *p* < 0.001). Attitudes and subjective norms were shown to have indirect effects of 0.144 and 0.309 on the actual proportion of physicians engaging in SDM behavior, while they respectively had 0.090 and 0.200 indirect effects on the actual role of medical decisions (Figure 1).

### Relationship of SDM Behavior Model Between Subgroups of Physicians

To explore whether the relationship of SDM behavior model varied between subgroups of physicians, gender, age, educational level, professional titles, working years, and training were included as covariates based on previous researches (Kaplan et al., 1996; Menear et al., 2018). Multiple Indicators Multiple Causes (MIMIC) model assessed the effects of covariates on factor structure. This model had a good fit (TLI = 0.98, CFI = 0.99, RMSEA = 0.05, WRMR = 0.81) to the data. Educational level, age, and receiving training regarding antibiotics were found to be significantly associated with physicians’ perception of SDM. Specifically, not receiving training regarding antibiotic prescribing had significantly slightly lower scores on physicians’ attitudes (*β* = −0.025, *p* < 0.001) and subjective norms of SDM (*β* = −0.019, *p* = 0.021). Female physicians (*β* = 0.173, *p* = 0.009) with higher educational level (*β* = 0.231, *p* = 0.003) had a higher intention of engaging in shared decision-making. The details of the effects of covariates on the SDM behavior model are presented in Table 3.

**TABLE3:** MIMIC model results of covariates on the SDM behavior model

Factor	Covariates effects on each other	*β*	SE	*p*
Attitude	Age	−0.008	0.006	0.162
	Gender	−0.016	0.087	0.858
	Professional titles	0.067	0.072	0.355
	Educational level	0.090	0.110	0.412
	Training	-0.025	0.007	<0.001
	Working years	0.001	0.003	0.717
Subjective norms	Age	−0.011	0.006	0.053
	Gender	0.038	0.087	0.655
	Professional titles	0.039	0.069	0.556
	Educational level	0.002	0.107	0.980
	Training	−0.019	0.008	0.021
	Working years	0.001	0.002	0.536
Perceived behavior control	Age	0.002	0.004	0.956
	Gender	−0.063	0.080	0.433
	Professional titles	0.083	0.062	0.176
	Educational level	0.102	0.101	0.309
	Training	0.004	0.006	0.463
	Working years	0.001	0.004	0.956
Behavior intention of SDM	Age	0.003	0.005	0.590
	Gender	0.173	0.050	0.009
	Professional titles	0.008	0.049	0.868
	Educational level	0.231	0.083	0.003
	Training	0.002	0.009	0.827
	Working years	−0.001	0.003	0.733

### Barriers to the Implementation of SDM

The most frequently reported barriers were the physicians’ perceived patients’ expectations (*n* = 398) and patients’ incapability of making the decision (e.g., insufficient health literacy, the complexity of the medical problem) (*n* = 321). In addition, lack of time (21.0%) and lack of training of SDM (18.8%) were also reported. Also, 7.3% of physicians also proposed other barriers outside the given options, such as the discordance of clinical guidelines and patients’ expectations, the obstinacy of patients with medical decisions (e.g., patients’ strong demand of antibiotics), and patients’ uncertainty with medical decisions and distrust between physicians and patients (Table 4).

**TABLE 4:** Physicians’ barriers to the implementation of shared decision-making

Barriers	N (%)^a^
Patients ask me to make decisions for them	398 (64.5)
It is too complicated for patients to make decisions	321 (52.0)
It is my job to make decisions for patients	270 (34.0)
Lack of time	129 (21.0)
Lack of training	116 (18.8)
It makes me feel uncomfortable to make shared decision with patients	39 (6.3)
Others**	45 (7.3)

a As each respondent was asked to rate one or more barriers, the overall percentage of all barriers was over 100%. **Represented the responses outside the given options, the obstinacy of patients with medical decisions (e.g., patients’ strong demand of antibiotics), distrust between physicians and patients, patients’ uncertainty with medical decisions, and discordance of clinical guidelines and patients’ expectations.

## Discussion

### Main Findings

Our study served as one of the first series of studies to understand physicians’ SDM behavior based on TPB theory and identified barriers to the implementation of SDM with patients presenting with ARIs in primary care in China. Based on the well-fitting structural equation model, our study revealed that attitudes and subjective norms of SDM were identified as significant predictors of behavior intention, and behavior intention was one significant predictor of physicians’ SDM behavior. However, physicians’ perceived behavior control was not a significant predictor of SDM behavior intention and SDM behavior. Physicians’ perceived patients’ expectations, patients’ incapability of making the decisions, and lack of time and training were identified as main barriers.

Consistent with the result of Philippe et al.’s (2015) systematic review that subjective norms was most frequently and significantly associated with behavior intention, our study demonstrated the most significant effects of physicians’ subjective norms on SDM behavior intention (Thompson-Leduc et al., 2015). Subjective norm refers to the influence of the social environment (e.g., pressure from peers, patients, or administrators) on physicians. The influence of patients may explain, at least in part, the significant effects of subjective norms on physicians’ intention of SDM in primary care.

As the relationship between the physicians and the patients is the most fundamental unit of decision-making, this interpersonal and interdependent rapport between physicians and patients may help explain the significant positive effects of subjective norms on primary care physicians’ intention of engaging in SDM. The interpersonal influence of patients would contribute to increasing the physicians’ intention of engaging in SDM, especially under the context of the shift from the traditional biomedical model to patient-centered care in recent decades (Mead and Bower, 2000; Greene et al., 2012; Thompson-Leduc et al., 2015).

Congruent with previous studies, the increased intention of engaging in SDM was also determined by their attitudes toward SDM (Thompson-Leduc et al., 2015). One recent research showed physicians’ attitudes (being favorably or unfavorably disposed toward the behavior) significantly predicted their intention of participating in medical education programs (Rich et al., 2020). This seems to suggest exposing physicians to an SDM training program might improve their intention of engaging in SDM.

It is worthy to note that whether physicians received training regarding antibiotics was one significant predictor of physicians’ “attitudes” and “subjective norms” with patients presenting with ARIs. It seems plausible that increasing clinical knowledge would help increase physicians’ confidence and mitigate physician’s uncertainty of medical decisions, which was identified as one important contributor of the physician’s reluctance to participate in SDM, especially in primary care facilities with limited medical resources (Politi and Street, 2011; Bae, 2017).

As for the effects of educational level and gender of physicians on SDM perception of SDM behavior, previous researches have demonstrated that training and education played an important role in SDM (Chen et al., 2016), and female physicians were more likely to engage more in partnership with patients and more involved in medical encounters (Bertakis, 2009).

However, physicians’ perceived behavior control was not a significant predictor of SDM behavior intention and SDM behavior in our study. Existing researches applying TPB to understand physician’s SDM behavior have demonstrated the significant effects of perceived behavior control on behavior intention in oncology, mental health, or under another context (Sassen et al., 2011; Guerrier et al., 2013). Given the current lack of studies applying TPB in understanding physicians’ SDM behavior with patients presenting with ARIs in primary care, whether perceived behavior control is one significant predictor of behavior intention and SDM behavior remains unclear. One possible explanation is that physicians may perceive it is easy to engage in SDM; however, the perception of patients’ incapability of SDM in primary care may offset physicians’ willingness and intention to engage in SDM (Driever et al., 2020). Further researches are needed for exploring the potential factors affecting the association between perceived behavior control and SDM behavior.

In this study, we identified a range of barriers to the implementation of SDM corresponding with the literature (Légaré and Witteman, 2013; Driever et al., 2020). Over half of physicians rated the perception of patients’ expectations as delegates and patients’ incapable of making decisions as to the most frequent important barrier. As indicated in the NHS report, “patients don’t want shared decision making” was raised as one of the challenges of implementation of SDM. The preference of whether involving in medical decisions should be based on patients themselves, rather than presumption of physicians (Joseph-Williams et al., 2017).

On the other hand, patients’ inability to share in decision-making and insistent patient demand for antibiotics was also identified as main barriers to the implementation of SDM, which help provide implications for SDM training and educating the public (Mathijssen et al., 2020). For example, public campaigns that serve to educate the public about the use of antibiotics can be recommended. The reasons of “patients unable to participate in SDM” and “patients unwilling to participate in SDM” should be differentiated. Consistent with previous studies, lack of time and training was one of the main barriers in primary care and tertiary facilities (Huang et al., 2015).

There were also some limitations in this study. First, it relied on physician self-report outcome of the actual proportion of SDM behavior and thus may be at risk of social desirability bias (Boivin et al., 2008). However, relative to other source of information, such as audio and video recordings of physician–patient consultations, self-report outcomes are argued to overestimate the true proportion of SDM behavior (Gordon and Street., 2016). Second, as this study was conducted in primary care facilities in Hubei province in China, the results should be interpreted with caution with limited generalizability.

## Conclusion

In light of our results, since subjective norms and attitudes, and behavior intention toward SDM behavior were demonstrated as significant predictors of SDM behavior, therefore, these may be a promising focus of SDM interventions in primary care in China. Interventions of SDM knowledge and skill development, and strategies such as educational meetings, educational materials, techniques such as role-playing, and decision support tools can be adopted to promote the engagement of SDM targeting physicians. On the other hand, based on the results of potential barriers to implementation of SDM, encouraging physicians to ask patients’ preferences directly and improve patients’ decisional capacity may be one potential solution. Furthermore, our results also call for the interventions targeting patients, such as SDM education and training programs, clinical knowledge, and educating on what antibiotics are and how antibiotics resistance develop, and rational use of antibiotics that equips and empowers physicians to engage in SDM.

## Data Availability

The raw data supporting the conclusion of this article will be made available by the authors, without undue reservation.
