# Supplementary material for: Applying Theory of Planned Behavior to Understand Physicians’ Shared Decision-Making With Patients With Acute Respiratory Infections in Primary Care: A Cross-Sectional Study
Source: Front Pharmacol. 2022 Jan 27;12:785419. doi: 10.3389/fphar.2021.785419 (PMC8828912; doi:10.3389/fphar.2021.785419)
Supplement: Supplementary file 1 [file DataSheet2.PDF]

Supplementary file 2: Hypotheses of covariates and the TPB model based on Multiple Indicators Multiple Causes (MIMIC) model

H_{c1}: Physicians' age, gender, professional titles, educational level, receive training, and working years affected physicians' attitudes towards shared decision making (SDM).

H_{c2} Physicians' age, gender, professional titles, educational level, receive training, and working years affected physicians' subjective norms towards SDM.

H_{c3} Physicians' age, gender, professional titles, educational level, receive training, and working years affected physicians' perceived behavior control towards SDM.

H_{c4} Physicians' age, gender, professional titles, educational level, receive training, and working years affected physicians' behavior intention of engaging in SDM.

The hypotheses were presented in Figure S1.

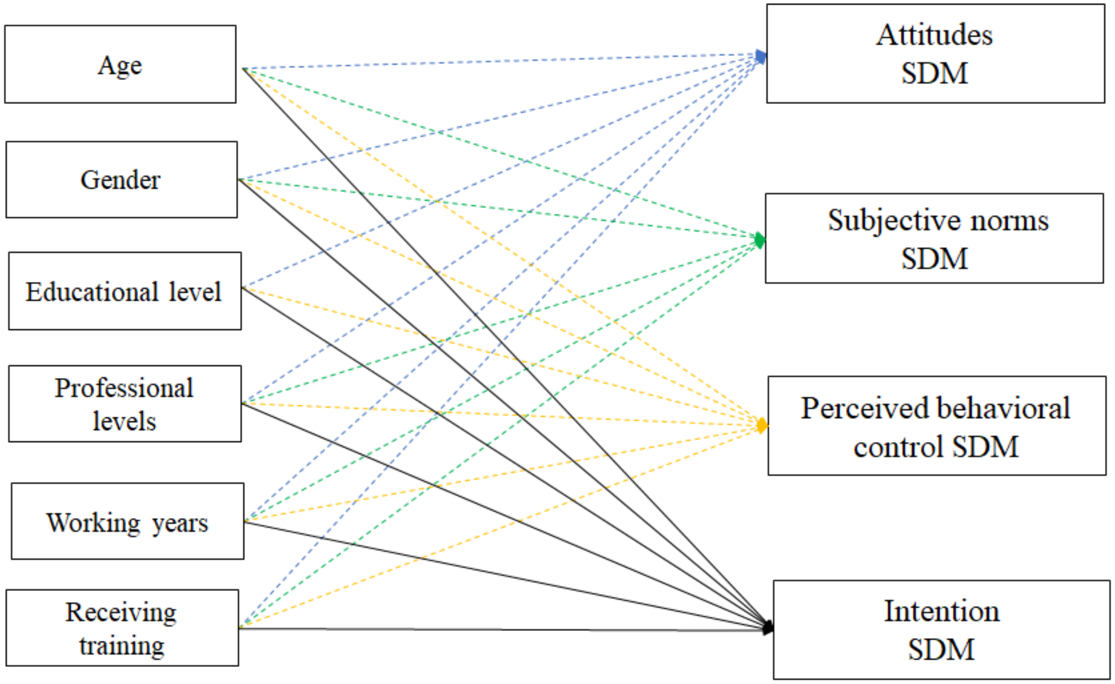

Figure S1. The Multiple Indicators Multiple Causes for shared decision-making with patients with ARIs

Note: Blue dashed lines-Hypotheses c1; Green dashed lines-Hypotheses c2;
Yellow dashed lines-Hypotheses c3; Black solid lines—Hypotheses c4.
