# Supplementary material for: Applying Theory of Planned Behavior to Understand Physicians’ Shared Decision-Making With Patients With Acute Respiratory Infections in Primary Care: A Cross-Sectional Study
Source: Front Pharmacol. 2022 Jan 27;12:785419. doi: 10.3389/fphar.2021.785419 (PMC8828912; doi:10.3389/fphar.2021.785419)
Supplement: Supplementary file 2 [file DataSheet1.PDF]

Supplementary file 1: List of items measuring physicians' perception of SDM based on TPB theory

When a treatment decision needs to be made during a consultation with a patient presenting with an acute respiratory infection (e.g. whether use antibiotics or not), what are your perceptions of shared decision making?

**Items**	**Response** (Likert-5 scale)
**Attitude of SDM**	
I think engaging in shared decision making would be___.	Very irresponsible–Very responsible
I think engaging in shared decision making would be___.	Very unpleasant–Very pleasant
I think engaging in shared decision making would make me feel___.	Worthless–Very worthwhile
**Subjective Norms of SDM**	
Most of the people who are important to me (e.g. peers, patients, mentors or administrators) would recommend that I engage in shared decision making.	Strongly disagree–Strongly agree
Most of the people who are important to me (e.g. peers, patients, mentors or administrators) would think it preferable that I engage in shared decision making.	Strongly disagree–Strongly agree
Most of the people who are important to me (e.g. peers, patients, mentors or administrators) are favorable that I engage in shared decision making.	Strongly disagree–Strongly agree
**Perceived Behavior Control of SDM**	
What are the chances that you can decide of engaging in SDM or not.	Very low–Very high
I feel I would be capable of engaging in shared decision making.	Strongly disagree–Strongly agree
I feel it is easy for me to engage in shared decision making.	Strongly disagree–Strongly agree
**Behavior Intention of SDM**	
I plan to engage in shared decision making.	Strongly disagree–Strongly agree

I intend to engage in shared decision making.	Strongly disagree–Strongly agree
